# Identification of Potential Biomarkers of Chronic Kidney Disease in Individuals with Diabetes: Protocol for a Cross-sectional Observational Study

**DOI:** 10.2196/16277

**Published:** 2020-07-31

**Authors:** Ashani R Lecamwasam, Mohammadreza Mohebbi, Elif I Ekinci, Karen M Dwyer, Richard Saffery

**Affiliations:** 1 Epigenetics Research Murdoch Children's Research Institute Victoria Australia; 2 Department of Endocrinology Austin Health Victoria Australia; 3 School of Medicine Faculty of Health Deakin University Victoria Australia; 4 Biostatistics Unit Faculty of Health Deakin University Victoria Australia; 5 Department of Medicine The University of Melbourne Victoria Australia; 6 Department of Paediatrics The University of Melbourne Victoria Australia

**Keywords:** epigenetics, metabolomics, gut microbiome, diabetes, chronic kidney disease

## Abstract

**Background:**

The importance of identifying people with diabetes and progressive kidney dysfunction relates to the excess morbidity and mortality of this group. Rates of cardiovascular disease are much higher in people with both diabetes and kidney dysfunction than in those with only one of these conditions. By the time these people are identified in current clinical practice, proteinuria and renal dysfunction are already established, limiting the effectiveness of therapeutic interventions. The identification of an epigenetic or blood metabolite signature or gut microbiome profile may identify those with diabetes at risk of progressive chronic kidney disease, in turn providing targeted intervention to improve patient outcomes.

**Objective:**

This study aims to identify potential biomarkers in people with diabetes and chronic kidney disease (CKD) associated with progressive renal injury and to distinguish between stages of chronic kidney disease. Three sources of biomarkers will be explored, including DNA methylation profiles in blood lymphocytes, the metabolomic profile of blood-derived plasma and urine, and the gut microbiome.

**Methods:**

The cross-sectional study recruited 121 people with diabetes and varying stages (stages 1-5) of chronic kidney disease. Single-point data collection included blood, urine, and fecal samples in addition to clinical data such as anthropometric measurements and biochemical parameters. Additional information obtained from medical records included patient demographics, medical comorbidities, and medications.

**Results:**

Data collection commenced in January 2018 and was completed in June 2018. At the time of submission, 121 patients had been recruited, and 119 samples remained after quality control. There were 83 participants in the early diabetes-associated CKD group with a mean estimated glomerular filtration rate (eGFR) of 61.2 mL/min/1.73 m2 (early CKD group consisting of stage 1, 2, and 3a CKD), and 36 participants in the late diabetic CKD group with a mean eGFR of 23.9 mL/min/1.73 m2 (late CKD group, consisting of stage 3b, 4, and 5), *P*<.001. We have successfully obtained DNA for methylation and microbiome analyses using the biospecimens collected via this protocol and are currently analyzing these results together with the metabolome of this cohort of individuals with diabetic CKD.

**Conclusions:**

Recent advances have improved our understanding of the epigenome, metabolomics, and the influence of the gut microbiome on the incidence of diseases such as cancers, particularly those related to environmental exposures. However, there is a paucity of literature surrounding these influencers in renal disease. This study will provide insight into the fundamental understanding of the pathophysiology of CKD in individuals with diabetes, especially in novel areas such as epigenetics, metabolomics, and the kidney-gut axis.

**International Registered Report Identifier (IRRID):**

DERR1-10.2196/16277

## Introduction

In 2011-2012, an estimated 1.7 million Australian adults had clinical and biochemical features of Chronic Kidney Disease (CKD), with similar numbers of males and females affected [1]. As kidney disease is mostly asymptomatic, the majority of people are unaware they have this chronic condition. Therefore, opportunistic testing in people with identifiable risk factors is of paramount significance to the individual’s health and Australia’s health economy. One of the leading risk factors for CKD is diabetes mellitus (DM), both type I and type II, which, together with associated micro and macrovascular complications, have reached epidemic proportions in Australia [2]. The prevalence of CKD is about three times higher in those with diabetes compared to those without [3]. One of the major microvascular complications of diabetes is kidney injury, termed diabetic chronic kidney disease. It is characterized by persistent albuminuria, proteinuria, and eventual decline in kidney function (estimated glomerular filtration rate of less than 60 mL/min/1.73 m^2^).

Among people with diabetes and CKD, the rate of cardiovascular events is more than twice the rate of those with diabetes alone [4]**.** Cardiovascular causes are the leading cause of mortality in people with diabetes and kidney disease, and this is more likely than the progression to end-stage renal disease (ESRD) [5]. These problems are only projected to escalate, given the growing epidemic of diabetes and obesity in Australia and worldwide.

Epigenetics is the study of a range of biochemical processes that regulate gene expression and phenotype in the absence of underlying alterations to the DNA sequence [6]**.** Epigenetic mechanisms play a crucial role in differentiation, cell specification, and function and may be regulated by external cues such as exposure to environmental pollutants and poor dietary choices. Consequently, this can induce abnormal metabolic phenotypes that can be further compounded by genetic susceptibility [7]**.** DNA methylation (DNAm) is the most studied epigenetic marker and is highly stable due to the covalent link to the underlying DNA. DNA methylation usually occurs at 5′-cytosines (5mC) of CpG dinucleotides. The regions of DNA with a higher number of CpG clusters are designated “CpG islands” and are generally methylated in a tissue-specific manner. Low methylation status of promoter CpG islands is associated with gene expression, while a high methylation status causes repression of transcription [8]**.**

Multiple factors such as inflammation, accelerated oxidative stress, accumulation of toxins, and aberrant metabolism are involved in the progressive deterioration of kidney function. Abnormal epigenetic mechanisms may be involved in mediating the likely gene-environment interactions underlying diabetes and chronic kidney disease [9]. This area of research is novel as we now know that pro-inflammatory and pro-fibrotic genes can be regulated by hyperglycemia via epigenetic mechanisms in vascular cells, monocytes, and mesangial cells [10]. The epigenetic mechanisms involved in the regulation of gene expression, including DNA methylation, appear to play a pivotal role in the development of diabetes-associated complications [11].

Metabolomics is the large-scale study of small molecules referred to as metabolites (such as sugars, amino acids, and lipids) in a given organism. Just as each individual will have a unique epigenetic profile, each will also have a characteristic metabolomic profile, leading to the concept of personalized metabolomics. In the future, this may provide the ability to track the trends of individual metabolomes over time, thus enabling personalized drugs and improved treatment strategies. Such personalized treatment is likely to be more effective than current medical population-based approaches. Metabolomic approaches are particularly promising in nephrology research as a consequence of the significant and varied impact kidney function has on circulating metabolite levels and because the metabolites may themselves play functional roles in CKD pathogenesis and its complications [12]. In experimental studies, metabolomics has been used to identify a signature of decreased mitochondrial function in diabetic chronic kidney disease, and these studies have outlined new therapeutic options [12].

Each individual’s microbiome composition is thought to be unique and influenced by genetics, geographical location, diet, age, and exposure to antibiotics, in addition to factors operational in early life such as mode of delivery and nature of early feeding. Gut bacteria play a crucial role in food digestion and nutrient absorption. More recently, the role of the gut in modulating the immune system has been recognized, and dysbiosis has subsequently been linked to an increasing number of non-communicable diseases such as diabetes, obesity, and heart disease.

Kidney disease is associated with inadequate nutrition, frequent use of antibiotics, metabolic acidosis, and volume overload. These factors are associated with microbial dysbiosis and may also affect gastrointestinal permeability, which together may account for the systemic inflammation that is associated with and contributes to worsening CKD and cardiovascular disease. CKD alters gut microbiota and contributes to dysbiosis. Vaziri et al reported altered gut microbiota composition in people with CKD: specifically, they noted lower numbers of *Lactobacillaceae* and *Prevotellaceae* families and 100 times higher *Enterobacteria* and *Enterococci* species [13].

The primary aim of this study is to compare DNA methylation, blood and urinary metabolomic, and gut microbiome profiles between people with diabetes and various stages of CKD. A component of this aim is to determine whether there are distinct profiles at each stage of diabetic CKD.

## Methods

A sample size of 120 provides 80% power to detect a minimum correlation of 0.25 between epigenetic/metabolomics and gut microbiome factors and the stage of diabetic kidney disease using a two-sided hypothesis test with a significance level of .05. A correlation of 0.25 is considered a moderately small effect size, and as such, the target sample size has enough power to investigate the primary research question.

The sample size of 120 will also provide more than 90% power to detect an R^2^ effect size of 0.20 (moderately small effect size) in a multivariate linear regression setting using an F test with a significance level (alpha) of 0.05.

This cross-sectional study design included a study population of 121 adults with diabetes and CKD stages 1, 2, 3a, 3b, 4, and 5. Participants were recruited from a single site, the Austin Health outpatient diabetes clinic, Victoria, Australia. Approximately 40 patients attend this clinic per week, and it took 6 months to recruit 121 participants. Patient recruitment commenced in January 2018 and was completed by the end of June 2018. Patients who presented to this clinic were offered the option to participate in the study and provided consent to the collection of clinical information, archiving and use of blood, urine, and stool samples, for research into the complications of diabetes.

All biological samples and data were de-identified and assigned a study number at Austin Health. Samples (other than stool samples) were transported from Austin Health to the Murdoch Children’s Research Institute (MCRI), Melbourne, for processing, analysis, and storage. Stool samples were transported on dry ice to the Metabolic Research Unit, Deakin University, Geelong for processing, analysis, and storage. Electronic data will be kept indefinitely to allow for continued analyses.

### Study Population and Recruitment

Participants (N=121) were recruited from the Austin Health outpatient diabetes clinic. The principal investigator approached patients while they waited for their appointment. The aims of the study were explained, and they were asked if they had an interest in knowing more information about the study. Those interested were provided with a participant information statement and consent form as well as a stool collection kit to be brought to their next diabetes clinic appointment.

### Inclusion and Exclusion Criteria

Participants qualified for inclusion if they were aged ≥18 years and diagnosed with diabetes and CKD stages 1, 2, 3a, 3b, 4, or 5.

Participants were excluded if they were aged <18 years, had a history of renal transplant, a single kidney, diabetes secondary to pancreatic pathology, steroid medication-induced diabetes, presence of non-diabetic kidney disease, active drug or heavy alcohol use, an active malignancy within the past five years, inflammatory bowel disease, were pregnant or breastfeeding, or who had a BMI <20 or >40.

### Data Collection

#### Patient Information

Participant data inclusive of age, gender, height weight, blood pressure, medical comorbidities, duration of diabetes, stage of CKD and its associated complications, medications, and pathology results were collected. The anthropometric data were obtained on the day of the clinic visit while the remainder of the patient’s information was gathered via access to Austin Health’s electronic medical records. All of this selected information was then entered into the study database.

#### Sample Storage

##### Serum/Plasma samples

Peripheral blood was collected at each outpatient clinic visit by venepuncture for assessment of epigenetics and metabolomics profiles. A total of 15mL of blood was collected at each visit: 10 mL in a 10-mL coagulant tube (for serum) and 5 mL in a 5-mL ethylene diamine tetraacetic acid (EDTA) anticoagulant tube (plasma, white blood cells). Samples were transported to the Austin Health laboratory within two hours of collection for processing by the principal investigator. The clot was initially separated from the 10ml coagulant tube. Subsequently, the 10-mL coagulant tube (for serum) was centrifuged at 3500 rcf at 4°C. The serum was separated into 0.5-mL aliquots. The clot and serum aliquots were then stored at –80°C. The 5-mL EDTA anticoagulant tube was also centrifuged at 3500 rcf at 4°C. The resultant plasma was separated into 0.5-mL aliquots, and the buffy coat separated into 0.2-mL aliquots. All samples were stored at –80°C.

##### Urine Samples

A spot urine sample was collected at each outpatient diabetic clinic visit and transported to the laboratory within 24-48 hours of collection. The samples were centrifuged at 3500 rcf at 4°C and then aliquoted into 5-mL tubes and stored at –80**°**C within 30 mins of processing. Subsequently, the urine and plasma samples were sent to Nightingale (Finland) for metabolomic biomarker analysis.

##### Stool Samples

Following collection in a specimen container, the samples were aliquoted into smaller 1.5-mL Eppendorf tubes before freezing at –80**°**C, then stored for DNA extraction to avoid multiple freeze/thaw cycles. The stool sample volume required for microbiome analysis was about 0.5-1.0 g.

### Data Generation

DNA methylation profile: Genomic DNA was extracted from blood lymphocytes (buffy coat) for methylation analysis. Peripheral blood was collected from Austin Health and transported to MCRI. Buffy coats were lysed with proteinase K for 2 hours, and the DNA was extracted using the Qiagen QIAamp DNA Mini spin kit (Ref 51306) according to the manufacturer’s protocol. DNA was quantified, and purification assessed by Qubit fluorometric quantitation (Thermo Fisher).

Genomic DNA (1000 ng) from adult buffy coat samples were randomized into 96-well plates and sent to the Australian Genome Research Facility (AGRF, Victoria) for sodium bisulfite treatment and genome-wide methylation analysis using Illumina InfiniumMethylationEPIC BeadChips (HM850K) [14]. The EPIC array measures DNA methylation at more than 850,000 CpG sites and covers all gene promoters, gene bodies, and ENCODE-assigned distal regulatory elements (Encyclopedia of DNA elements) [15]. Quality assessment was performed by QuantiFluor, and a subset of samples was resolved on a 0.8% agarose gel at 130 V for 60 minutes. Samples were then normalized to approximately 500 ng of DNA in 45 μL and bisulfite converted with the Zymo EZ DNA Methylation kit. All samples were above 860,000 detected CpG sites (*P*<.01). Raw IDAT files were received on a hard disk from AGRF and used for data analysis.

In order to assess blood and urine metabolites, plasma was isolated from peripheral blood and corresponding urine samples sent to Nightingale (Finland) for metabolomic biomarker analysis. This platform analyzes metabolites using nuclear magnetic resonance (NMR) spectroscopy, which is an NMR-based metabolomics platform [16]. Robotic sample preparation is followed by spectral acquisition in a fully automated manner. The NMR platform has been used to profile approximately 350,000 blood samples in over 1000 epidemiological and clinical studies. The biomarker measurements are acquired from native serum or EDTA plasma and are possible with 100 μL to 350 μL sample volume. The platform provides quantification of 228 metabolic measures, which are quantified in absolute concentrations (ie, mmol/L) [16].

In order to assess the gut microbiome, stool samples were sent on dry ice to the Metabolic Research Unit, Deakin University, Geelong, where they were stored in a –80°C freezer. DNA was extracted using the commercial Qiagen QIAamp DNA Stool Mini Kit (Ref 51504) according to the manufacturer’s protocol. DNA quantity and purity were assessed using Qubit (Thermo Fisher).

The Australian Genome Research Facility performed PCR amplification and sequencing. PCR amplicons were generated using the primers and conditions outlined in Table 1.

Thermocycling was performed on an Applied Biosystem 384 Veriti using AmpliTaq Gold 360 Mastermix (Life Technologies, Australia) for the primary PCR. The first stage PCR product was purified using magnetic beads, and samples were visualized by electrophoretic separation in a 2% Sybr Egel (Thermo Fisher). A secondary PCR to index the amplicons was performed with TaKaRa Taq DNA Polymerase (Clontech). The resulting amplicons were purified using magnetic beads, quantified by fluorometry (Promega Quantifluor), and normalized. The equimolar pool was purified a final time using magnetic beads to concentrate the pool and then measured using a High-Sensitivity D1000 Tape on an Agilent 2200 TapeStation. The pool was diluted to 5 nM, and molarity was confirmed using a High-Sensitivity D1000 Tape, then sequenced on an Illumina MiSeq with a V3, 600 cycle kit (2 × 300 base pairs paired-end).

Paired-end reads were assembled by aligning the forward and reverse reads using PEAR (version 0.9.5) [17]. Primers were identified & trimmed. Trimmed sequences were processed using Quantitative Insights into Microbial Ecology (QIIME 1.8) [18], USEARCH (version 7.1.1090) [19,20], and UPARSE [21] software. USEARCH sequences were quality filtered, and full-length duplicate sequences were removed and sorted by abundance. Singletons or unique reads in the data set were discarded. Sequences were clustered and chimera filtered using the “rdp_gold” database as the reference. Reads were mapped back to OTUs with a minimum identity of 97% to obtain the number of reads in each OTU. QIIME taxonomy was assigned using the Greengenes database (version 13_8) [22].

Demographic and clinical information stored on the Austin Health patient database was accessed and recorded in the study database. The information included but was not limited to blood pressure, weight and BMI, eGFR, albumin to creatinine ratio, medical comorbidities, and medication history. This information was de-identified and given a study ID number corresponding to their matched biological samples.

**Table 1:** Primers and conditions used to amplify the 16S: V3-V4 target sequence.

Primers and conditions		Description
**Primer**		
	Target	341F-806R
	Forward (341F)	CCTAYGGGRBGCASCAG
	Reverse (806R)	GGACTACNNGGGTATCTAAT
**Condition**		
	Target	16S: V3 - V4
	Cycle	29
	Initial	95°C for 7 min
	Disassociate	94°C for 60 sec
	Anneal	50°C for 60 sec
	Extension	72°C for 60 sec
	Finish	72°C for 7 min

## Results

This study was approved in July 2017 by the Human Research Ethics Committee of Austin Health, Victoria, Australia (HREC/17/Austin/166) and Deakin University, Geelong, Australia. The study was funded in July 2017. Data will be published in peer-reviewed medical and scientific research journals and any molecular data published in the appropriate public repositories. Data collection commenced in January 2018 and was completed in June 2018. At the time of this submission, 121 patients had been recruited. After sample quality control, data were available for 119 patient samples. The proportion of the 119 recruited patients in each stage of CKD is illustrated in Table 2. The clinical and biochemical characteristics of our patient cohort are shown in Table 3.

The majority of participants had type 2 diabetes (n= 99), while 20 had type 1 diabetes. Only 2 of the 20 patients with type 1 diabetes were characterized as having latent autoimmune diabetes of adulthood (LADA). There were 83 participants in the early diabetes-associated CKD group with a mean eGFR of 61.2 mL/min/1.73 m^2^ (early CKD group consisting of stages 1, 2, and 3a), and 36 participants in the late diabetic CKD group with a mean eGFR of 23.9 mL/min/1.73 m^2^ (late CKD group, consisting of stages 3b, 4, and 5) (*P*<.001). We chose to define late diabetes-associated CKD as Stages 3b, 4, and 5 in recognition of the marked increase in death, cardiovascular events, and hospitalizations observed as eGFR decreased below 45 mL/min/1.73 m^2^ [23]. The mean age in the early CKD group was significantly younger at 66.1 years versus 72 years in the late CKD group (*P*=.01). There was a higher proportion of males, with 50 out of 83 participants (60.2%) in the early CKD group versus only 16 males out of 36 participants (44.4%) in the late CKD group.

Biospecimens are currently being used for epigenetic, metabolomic, and gut microbiome analyses. The results from these respective analyses will be completed in June 2020 with the publication of this work expected later this year.

**Table 2:** The proportion of patients in each stage of chronic kidney disease (N=119).

Disease stage	Patients, n (%)
Stage 1	8 (7)
Stage 2	49 (41)
Stage 3a	26 (22)
Stage 3b	13 (11)
Stage 4	13 (11)
Stage 5	10 (8)

**Table 3:** Clinical and biochemical characteristics of the patient cohort.

Patient characteristics		Early chronic kidney disease (group 1)	Late chronic kidney disease (group 2)	*P* value
Age (years), mean (SD)		66.14 (11.5)	72.00 (11.5)	.01
Male, n (%)		50 (60.2)	16.00 (44.4)	.16
**Type of diabetes, n (%)**				.24
	Type 1	15 (18.1)	3 (8.3)	
	Type 2	66 (79.5)	33 (91.7)	
	LADA^a^	2 (2.4)	0 (0.0)	
Duration of DBM^b^ (years), mean (SD)		18.71 (11.0)	33.00 (11.2)	.12
Hypertension, n (%)		65 (78.3)	34 (94.4)	.06
Diabetic retinopathy, n (%)		32 (38.6)	15 (41.7)	.91
Cardiovascular disease, n (%)		30 (36.1)	15 (41.7)	.72
Peripheral vascular disease, n (%)		12 (14.5)	10 (27.8)	.14
Dyslipidemia, n (%)		66 (79.5)	31 (86.1)	.55
Depression, n (%)		16 (19.3)	4 (11.1)	.41
**Smoking status, n (%)**				.51
	Nonsmoker	46 (55.0)	24 (66.7)	
	Ex-smoker	30 (36.0)	10 (27.8)	
	Current smoker	7 (8.4)	2 (5.6)	
BMI (kg/m^2^), mean (SD)		29.44 (7.9)	28.53 (7.9)	.58
SBP^c^ (mmHg), mean (SD)		109.58 (49.7)	121.09 (50.0)	.26
DBP^d^ (mmHg), mean (SD)		63.38 (27.2)	66.20 (20.1)	.59
Hb^e^ (g/L), mean (SD)		108.60 (52.3)	88.75 (53.7)	.06
eGFR^f^ (mL/min/1.73 m^2^), mean (SD)		61.17 (22.8)	23.89 (12.0)	<.001
HbA_1c_^g^ (%), mean (SD)		7.51 (1.8)	7.66 (1.7)	.69
TC^h^ (mmol/L), mean (SD)		4.00 (1.1)	3.74 (1.0)	.25
LDL^i^ (mmol/L), mean (SD)		1.89 (0.9)	1.78 (0.9)	.54
Urine albumin/creatinine ratio, mean (SD)		20.57 (73.27)	83.71 (178.06)	.28
Urine protein/creatinine ratio, mean (SD)		0.28 (1.57)	4.15 (13.85)	.35

^a^LADA: latent autoimmune diabetes of adulthood.

^b^DBM: diabetes mellitus.

^c^SBP: systolic blood pressure.

^d^DBP: diastolic blood pressure.

^e^Hb: hemoglobin.

^f^eGFR: estimated glomerular filtration rate.

^g^HbA_1c_: glycated hemoglobin.

^h^TC: total cholesterol.

^i^LDL: low-density lipoprotein.

## Discussion

In our preliminary data, we have shown the proportion of individuals with diabetes and various stages of CKD. We have illustrated the clinical and biochemical characteristics of our patient cohort. With this protocol, we have obtained DNA for methylation and microbiome analyses and are currently analyzing these results together with the metabolome of our patient group. There have been separate studies in the areas of epigenetics [24], metabolomics [25], and the gut microbiome [13] that have shown these biomarkers to be potential indicators of renal dysfunction and markers of renal prognosis. However, no studies have simultaneously investigated the possible combined roles of epigenetics, metabolomics, and gut microbiome, especially across all stages of chronic kidney disease in individuals with diabetes.

One of the strengths of this study protocol is the depth of cross-sectional data across epigenetics, metabolomics, and the gut microbiome as well as varying biospecimens inclusive of serum, plasma, buffy coats, urine, and fecal samples, involving the different stages of kidney disease. This broad scope will enable a comprehensive investigation of the factors contributing to and potential for biomarker identification in people with diabetes-associated CKD. One of the limitations, however, of this study design is its cross-sectional nature and small sample size, especially in the late CKD group. Future prospective cohort designs would necessitate larger sample sizes in each CKD stage as well as longitudinal data collection.

The significance and clinical value of these potential biomarkers are in determining whether the specific profiles across the three domains could help to predict the stages of renal dysfunction, especially if these are demonstrated to precede the change in cellular or clinical phenotype. This protocol provides the first step towards biomarker discovery for future longitudinal studies that would enable longer-term patient follow-up. Demonstrating such a change may lead to targeted, individualized patient treatment and better patient outcomes. There is a paucity of research exploring the clinical impact of epigenetics, metabolomics, and the gut microbiome in renal disease. Our research will generate data relating to epigenomic and metabolomic analyses, which, together with an understanding of the kidney-gut microbiome axis, will be a means of identifying potential novel biomarkers for people with progressive diabetic CKD.

## Abbreviations

AGRF: Australian Genome Research Facility
CKD: chronic kidney disease
DM: diabetes mellitus
DN: diabetic nephropathy
EDTA: ethylenediaminetetraacetic acid
eGFR: estimated glomerular filtration rate
ENCODE: Encyclopedia of DNA Elements
ESRD: end-stage renal disease
HREC: Human Research Ethics Committee
LDL: low-density lipoprotein
MCRI: Murdoch Children’s Research Institute
NMR: nuclear magnetic resonance
RRT: renal replacement therapy
